# 

**Published:** 1882-10

**Authors:** 


### BOOKS AND PERIODICALS.

THE STUDENTS' MANUAL OF HISTOLOGY, for the use of students practitioners and microscopists—Second Edition—By Chas. W. Stowell, M. D.

Dr. Stowell's manual is an excellent one. It is the result of a large experience in teaching histology to a class of four hundred or more pupils, in the University of Michigan. This of itself would be sufficient qualification for writing a manual. We commend Professor Stowell's Histology to students and physicians who are beginning the study, as an invaluable aid—we know of nothing better. Price, $2.50. George S. Davis, Medical Publisher, Detroit, Mich.

"WHAT TO DO IN CASES OF POISONING."—This is the title of a neat little volume, by William Murrell, M. D., M. R. C. P., upon a subject clearly set forth in its title. A very useful and handy little book for the "busy practitioner" to carry in his vest pocket. In it all poisons and their antidotes are alphabetically arranged, with short, crisp and reliable instructions how to proceed in all cases of poisoning. Published by Geo. S. Davis, Detroit.

"Ten Years Experience in the Treatment of Stricture of the Urethra by Electrolysis," by Robert Newman, M. D. A reprint from the *Medical Record*. A pamphlet treating intelligently upon the subject stated. Author's address not given.

"Some of the Simple Methods of Performing Hystero-Trachelorrhaphy." Reprint from the *Obstric Gazette*. By O. E. Herrick, M. D., 52 Monroe street, Grand Rapids, Mich.

"The Physician Himself and what He Should add to His Scientific Acquirements," is the title of a most excellent book of advice to young physicians. It is written by Dr. D. W. Cathell, of Baltimore, and published by Cushing & Bailey, 262 West Baltimore street, at $1.25 a copy. It is well printed, neatly bound, while the instruction contained within its boards, to the physician anxious to obtain a lucrative practice, is certainly of the most wholesome and timely character. We commend the book most heartily.

"Stricture of the Rectum, treated by Electrolysis," by Robert Newman, M. D., of New York. A reprint from the *New England Medical Monthly*, is a valuable paper upon the subject indicated in its title. To be had of the author.

